# Effects of metformin phonophoresis and exercise therapy on pain, range of motion, and physical function in chronic knee osteoarthritis: randomized clinical trial

**DOI:** 10.1186/s13018-024-05120-0

**Published:** 2024-10-26

**Authors:** Marwah Salih Abed, Marihan Zakaria Aziz, Nabil Mohie AbdelHamid, Elsadat Saad Soliman

**Affiliations:** 1 Department of Physical Therapy for Musculoskeletal Disorders and its Surgeries, Faculty of Physical Therapy, Cairo University, Cairo, Egypt; 2 Department of Physical Therapy for Musculoskeletal Disorders and its Surgeries, Faculty of Physical Therapy, Cairo University, Giza, Egypt; 3 Faculty of Pharmacy, Kafrelsheikh University, Kafr Elsheikh, Egypt; 4 Department of Physical Therapy for Musculoskeletal Disorders, and its Surgeries, Faculty of Physical El Maadi, Cairo, Egypt

**Keywords:** Knee osteoarthritis, Exercise, Metformin Phonophoresis, Ultrasound

## Abstract

**Background:**

Knee osteoarthritis (KOA) is a common musculoskeletal disorder. Therapeutic ultrasound (US) is a safe and effective treatment for KOA. It relieves knee pain and enhances function. Metformin (MF) regulates chondrocytes, hence providing chondroprotection. Furthermore, it efficiently reduces knee articular cartilage degeneration and retards the progression of osteoarthritis. However, the localized administration of MF by phonophoresis for KOA has yet to be studied.

**Purpose:**

To assess the possible effects of metformin phonophoresis (MFPH) plus exercise therapy (EX) compared to MFPH alone or the US on knee pain, function, and range of motion (ROM) in chronic KOA patients.

**Methods:**

Seventy-eight patients with unilateral mild to moderate chronic KOA were included. Patients were randomly assigned to three groups: group A (MFPH + EX), group B (MFPH alone), and group C (US). The US group used an acoustic-neutral gel, while the MFPH group used a gel containing 1.2% MF. The exercises included hamstring stretches, calf stretches, and knee strengthening exercises. Treatment in the three groups continued for four weeks (three sessions per week). The Visual Analog Scale (VAS), the Western Ontario and McMaster Universities Osteoarthritis Index (WOMAC), and the goniometer were used to assess knee pain, function disability, and ROM, respectively. All measures were recorded before, **2** weeks, and **4** weeks after the intervention in all groups. Multivariate Analysis of Variance (MNOVA) was performed to compare the effects within and between groups for knee ROM and function disability. The Kruskal-Wallis test and the Friedman test analyzed the pain intensity.

**Results:**

When the baseline patient characteristics were compared, there were no significant differences in means of age, gender, body mass index (BMI), or lower limb dominance across the three groups (*p* > 0.05). After 4 weeks of intervention, clinical outcomes significantly improved in all three groups (*p* < 0.05). However, patients in the MFPH + EX group improved significantly in all outcomes compared to the MFPH and US groups (*p* < 0.05).

**Conclusion:**

Post-treatment results showed a statistically and clinically significant improvement in pain intensity, knee ROM, and function in the MFPH group; however, combining MFPH with exercises is more beneficial in reducing KOA symptoms.

**Trial registration:**

Clinical Trial Registry at (pactr.samrc.ac.za) database.

NO: PACTR202311507335269. Date: November 9, 2023 (retrospectively registered).

**Supplementary Information:**

The online version contains supplementary material available at 10.1186/s13018-024-05120-0.

## Introduction

Knee osteoarthritis (KOA) is a degenerative joint condition characterized by degeneration, which is commonly attributed to the cumulative effects of mechanical stress and the slow deterioration of articular cartilage [1]. KOA is a highly prevalent musculoskeletal disorder that affected 300 million people globally in 2017. It can affect any joint, although the most commonly affected are the knees, hands, hips, and spine [2].

The articular cartilage comprises chondrocytes and extracellular matrix (ECM). The progressive degradation of the ECM of the articular cartilage is one of the significant pathogenic events leading to wear and exposure of the subchondral bone, osteophyte formation, and eventually osteoarthritis **(**OA), which often causes disability and pain, particularly in individuals over 50 years of age [3]. Knee osteoarthritis is typically characterized by joint pain that worsens with physical activity but decreases with rest. Other symptoms include tenderness, joint stiffness, crepitus, limited ROM, joint effusion, deformity, and instability [4].

Knee osteoarthritis treatment recommendations often consist of pharmacological (analgesics, such as NSAIDs and intra-articular corticosteroid injections), non-pharmacological (physical therapy and patient education), and surgical interventions (if not conservatively treated). Most guidelines recommend core treatment for KOA, which includes education, exercises, and weight loss. However, these guidelines offer a sequential, phased approach for managing KOA beyond these treatments and co-morbidity group [5]. Exercise therapy is the first non-pharmacological and cornerstone treatment approved by international high-quality guidelines for patients with KOA [5, 6]. Exercises reduce pain, stiffness, joint dysfunction, and muscular weakness in individuals with KOA. These also prevent cartilage degeneration, inflammation, and subchondral and metaphyseal bone trabeculae loss [7]. Furthermore, exercise training has been demonstrated to promote cardiorespiratory function, muscle strength, postural stability, and psychological health, making it a necessary adjunct therapy for treating patients with KOA [8].

Therapeutic ultrasound (US) is a non-invasive approach for treating chronic KOA and is usually used in conjunction with other therapies, such as physical therapy, exercise therapy, medications, and lifestyle changes to improve effectiveness [9–11].

Phonophoresis is a commonly employed technique that augments the physical absorption of pharmaceuticals via US waves, making the skin more receptive to topical treatments [12]. In the past, a variety of drugs, including dexamethasone, piroxicam, and ibuprofen, were combined with phonophoresis and demonstrated a notable improvement in knee function and pain relief [12–15].

Metformin enhances mitochondrial function in OA chondrocytes, reducing pain and attenuating structural deterioration by activating adenosine monophosphate-activated protein kinase (AMPK), a crucial cellular energy sensor. This nutrient and energy sensor may treat or prevent OA, thus metformin offers pain reduction and cartilage preservation [16, 17]. Metformin has recently been used as an adjunct to surgical and non-surgical periodontal therapy to treat chronic periodontitis. When applied locally to the periodontal pocket, the 1% metformin gel showed superior drug-release patterns and clinical efficacy [18, 19]. However, few studies have been employed to determine the efficacy of oral anti-diabetic metformin in an injectable form into the knee joint to suppress bone metastases [20]. The local influence of metformin therapy on degenerative changes in chronic KOA has yet to be explored.

Recently, metformin has received increasing attention due to its potential anti-proliferative properties. Consequently, metformin has been tested for its efficacy in treating KOA in an in-vitro trial [21]. As a novel approach to treating KOA patients, limited research has assessed the local administration of metformin utilizing phonophoresis and its potential impact on osteoarthritic individuals. It proposes inhibiting intra-knee matrix metalloproteinase (MMPs) activity and relieving OA symptoms. Thus, the present study would grow evidence regarding the possible effects of MFPH as a standalone intervention and in combination with exercises, compared to therapeutic US on knee pain, ROM, and physical functions of KOA patients.

## Methods

### Participants

Seventy-eight male and female patients diagnosed with mild to moderate unilateral KOA participated in a parallel-group, randomized, single-blinded clinical trial. Orthopedists directly referred patients for recruitment, and they were assessed according to the American College of Rheumatology’s (ACR) standards for clinical and radiographic symptoms [22, 23]. Before the initiation of the study, each patient received information about the study’s details and signed a consent form. The study was approved by the Research Ethical Committee at the Faculty of Physical Therapy, Cairo University, Egypt (No. P.T.REC/012/004282), and it adhered to the ethical guidelines outlined in the 1964 Declaration of Helsinki [24]. The Pan African Clinical Trials Registry lists this trial (No. PACTR202311507335269).

The eligible patients met the following inclusion criteria: age 45 to 65 years, BMI 18.5–29.9 kg/m^2^, diagnosis of KOA according to American College of Rheumatology criteria [22, 23], and grade I-III in the Kellgren-Lawrence grading system [25, 26].

Patients were excluded if they had any of the following characteristics: rheumatologic conditions like rheumatoid arthritis; prior knee joint surgery; previous lower extremity fracture with knee joint involvement; diabetes; neuropathy and sensory disorders; intraarticular corticosteroids injection or platelet-rich plasma in the preceding six months; thrombosis of the lower limbs; any contraindications for the use of ultrasound (e.g., open epiphysis, infection, heart problems, metal implants, pacemaker, pregnancy), or thrombophlebitis; wound or laceration in the soft tissues surrounding the knee; skin disease; usage of painkillers during the study, which might have influenced the results; knee physiotherapy during the three months prior; or other medical procedures, such as aspiration and knee arthroplasty.

Patients were randomly assigned to three groups. Group A (MFPH plus EX group) underwent both MFPH and conventional exercises. Group B (MFPH) received MFPH. Group C (US group) received the US with an acoustic neutral gel (a non-drug standard coupling gel).

### The randomization and blinding

Patients were randomly assigned using a random generator (https://www.randomizer.org/) and remained blind to their group assignment. Before the start of the trial, the allocations were hidden in sequentially numbered, sealed, opaque envelopes. By ensuring that no patient was aware of the other treatment that the other group received, the allocation of patients to groups was concealed from them. The researcher conducted intervention sessions for each treatment group independently to guarantee the blinding process.

### The preparation of the gel

The metformin gel (medicinal coupling medium) was prepared with the assistance of Professor Abdel-Hamid M Nabil at the Faculty of Pharmacy, Kafr El-Sheikh University. Metformin gel 1% was prepared as described by Baldassari et al. [27]. [27]. All the required formulation ingredients were weighed accurately. Metformin HCl powder 34.5 g was dissolved in 20 ml glycerin. Then, the prepared solution was added gradually to 2850 g of the ultrasound gel with constant mixing to ensure homogeneity. Finally, 2.85 g of sodium citrate was added as a preservative, and the mixing continued until clear gel was obtained (10–15 min). Its color, appearance, and odor were quite like the coupling agent routinely used in the US. Lastly, the prepared gel was sealed in 30 g tubes and subsequently utilized to evaluate the clinical impacts.

### Outcomes measures

All demographic data, including age, gender, lower limb dominance, weight, and height, was collected, and the affected knee was targeted based on more symptoms or higher pain levels.

#### Knee pain

The Arabic version of the visual analog scale (VAS) was used to quantify the degree of knee pain throughout the previous week. It ranged from 0 (no pain) to 10 cm (worst pain). The VAS is reliable and valid for assessing knee pain intensity [28].

#### Physical function

The Western Ontario and McMaster Universities Osteoarthritis (WOMAC) index consists of three subscales: physical function, articular stiffness, and activity-related pain. The total scores range from 0 to 96 and indicate the severity of osteoarthritis. The WOMAC index values were evaluated using a Likert scale, with 0 indicating no pain, limitation, or dysfunction and 4 indicating significant pain or limitation [29]. The Arabic version of the WOMAC index is a valid and reliable instrument for assessing functional disability in KOA [30].

#### Knee range of motion

A smartphone application (Goniometer Records) is a valid and reliable tool for evaluating knee ROM and joint position sense [31, 32]. Each patient was instructed to assume a supine position for knee ROM measurements: (a) Knee extension: The patient was seated with the end of a plinth with a 90° flexed knee. The smartphone was then positioned below the head of the fibula by 2 cm with the phone screen facing away, and it was ensured that both were positioned. The longitudinal axes of the smartphone and fibula were parallel to each other. Then, the researcher pressed the start button to define the fixed arm of the smartphone goniometer. Then, the patient was asked to extend his/her knee from the resting position (90º) as much as he/she could, and then the researcher pressed the stop button and recorded the result. (b) Knee flexion: Each patient was lying supine and instructed to hold the knee and maintain an angle for taking all the measurements; a 90°-bent limb support would be attached to the table and positioned in the popliteal fossa. In the same position, each patient was asked to do maximal active flexion before the examiner pressed the stop record button. The average scores of the three trials were used to calculate the patient’s ROM score. There was a 2-minute break in between each knee flexion and extension measurement.

## Intervention

### Phonophoresis of metformin gel (MFPH)

In the MFPH group (Group B), a gel containing 1.2% metformin was topically applied to the medial and lateral aspects of the affected knee using a circular motion. Using a US device (manufactured by Zimmer Medizin Systeme GmbH), continuous US waves were administered at a frequency of 1 MHz, an intensity of 1 W/cm^2^, and a head total surface area of 5 cm^2^, each lasting 10 min. Before treatment, the patient’s skin was cleansed using cotton and alcohol.

#### Ultrasound therapy

In the US group (Group C), the US was applied using an acoustic neutral gel. The device’s parameters and application were similar to those of the MFPH group.

### Metformin phonophoresis and traditional exercise

**Fig. 1:**
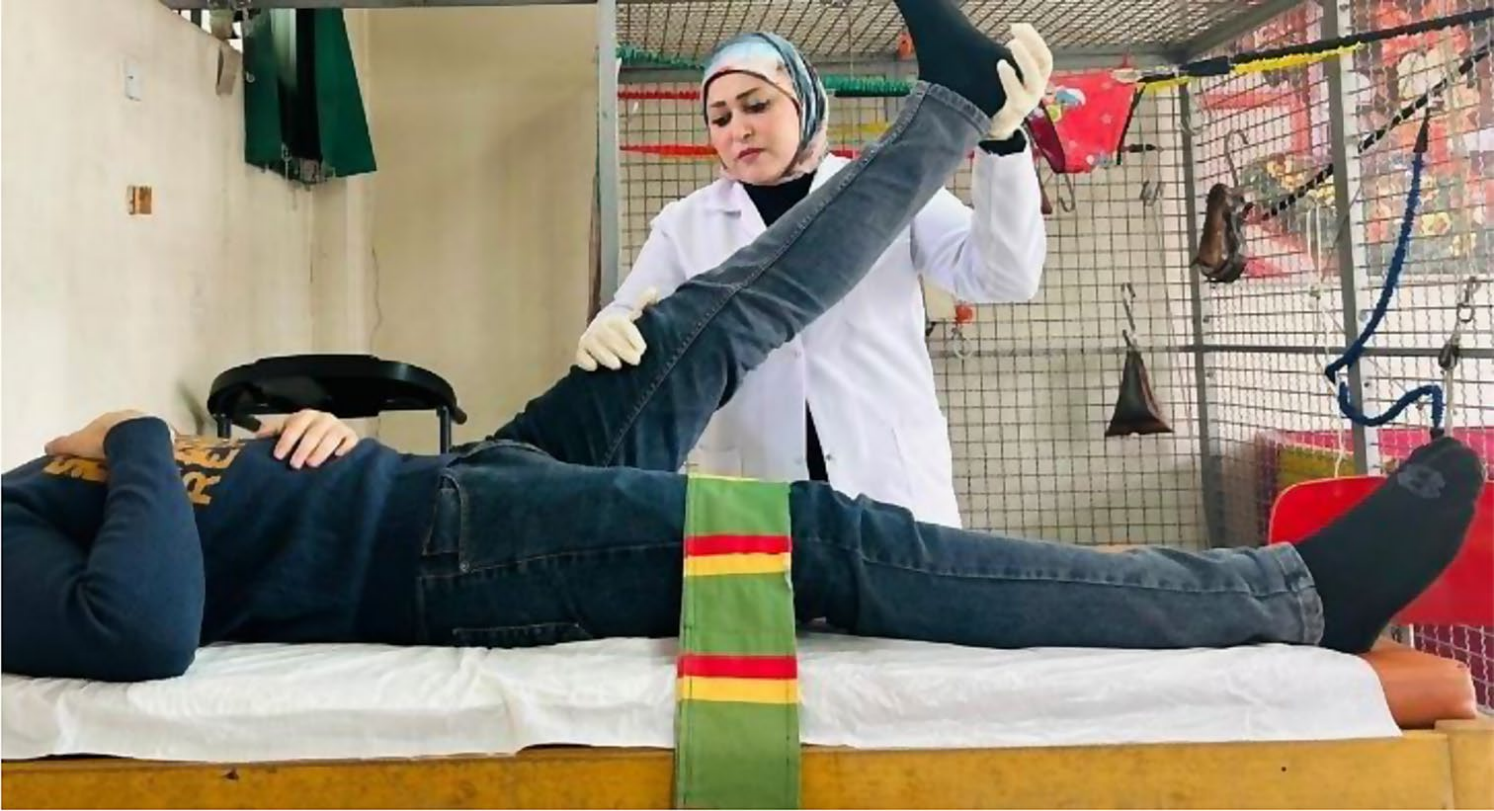
Stretching of hamstrings and calf muscles

**Fig. 2:**
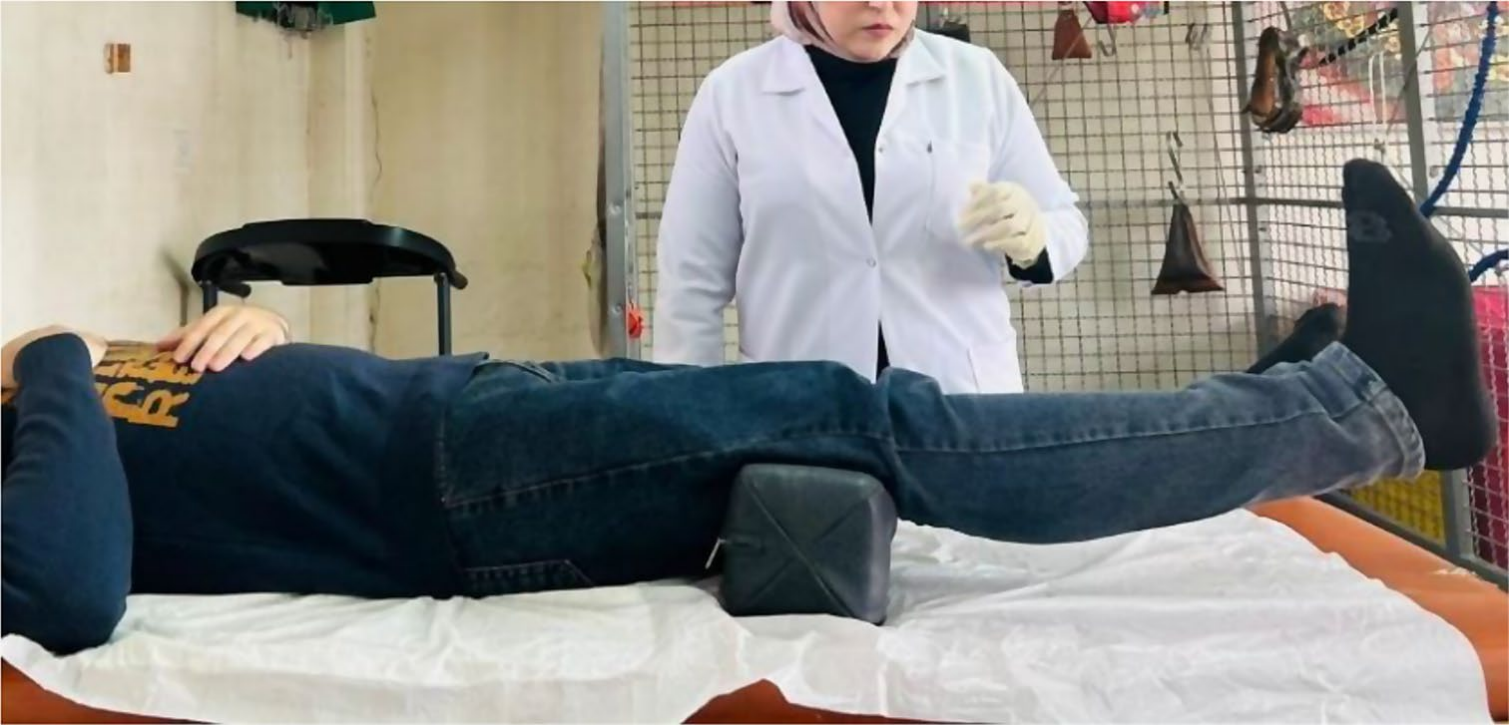
Terminal knee extension

**Fig. 3:**
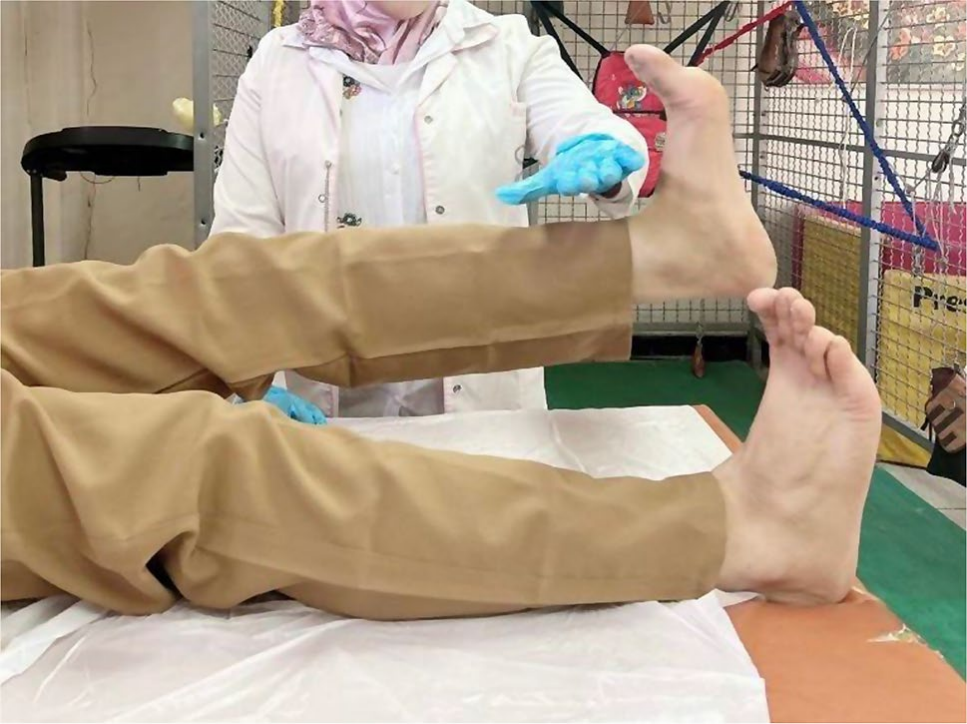
Straight leg raising from supine lying

**Fig. 4:**
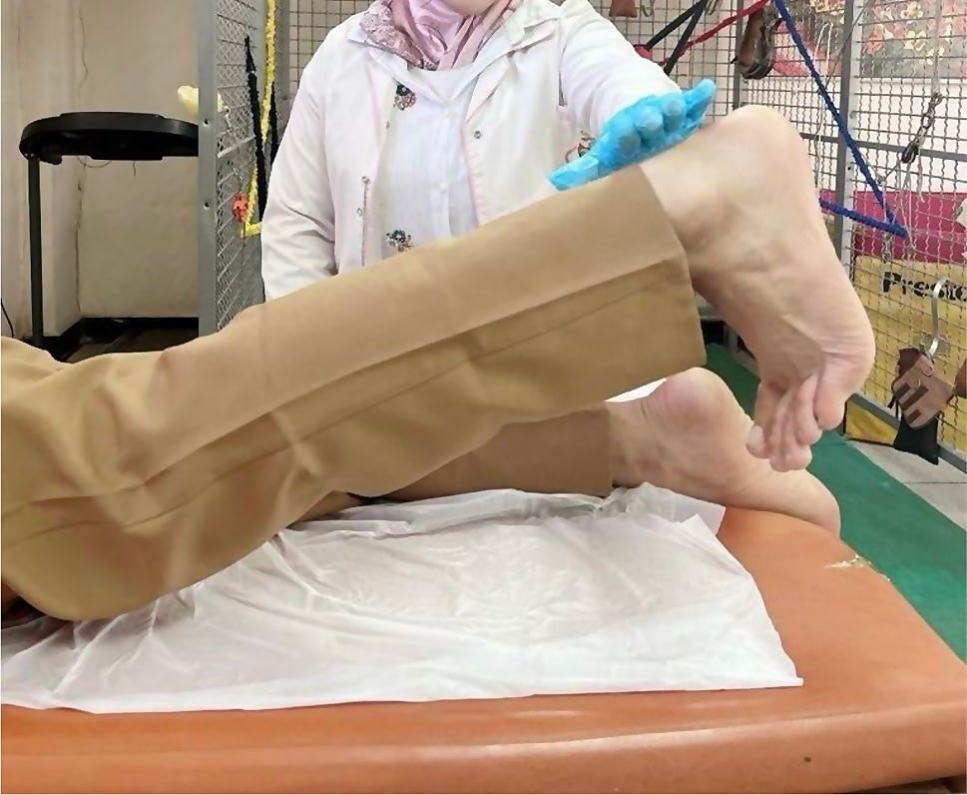
Straight leg raising from prone lying

**Fig. 5:**
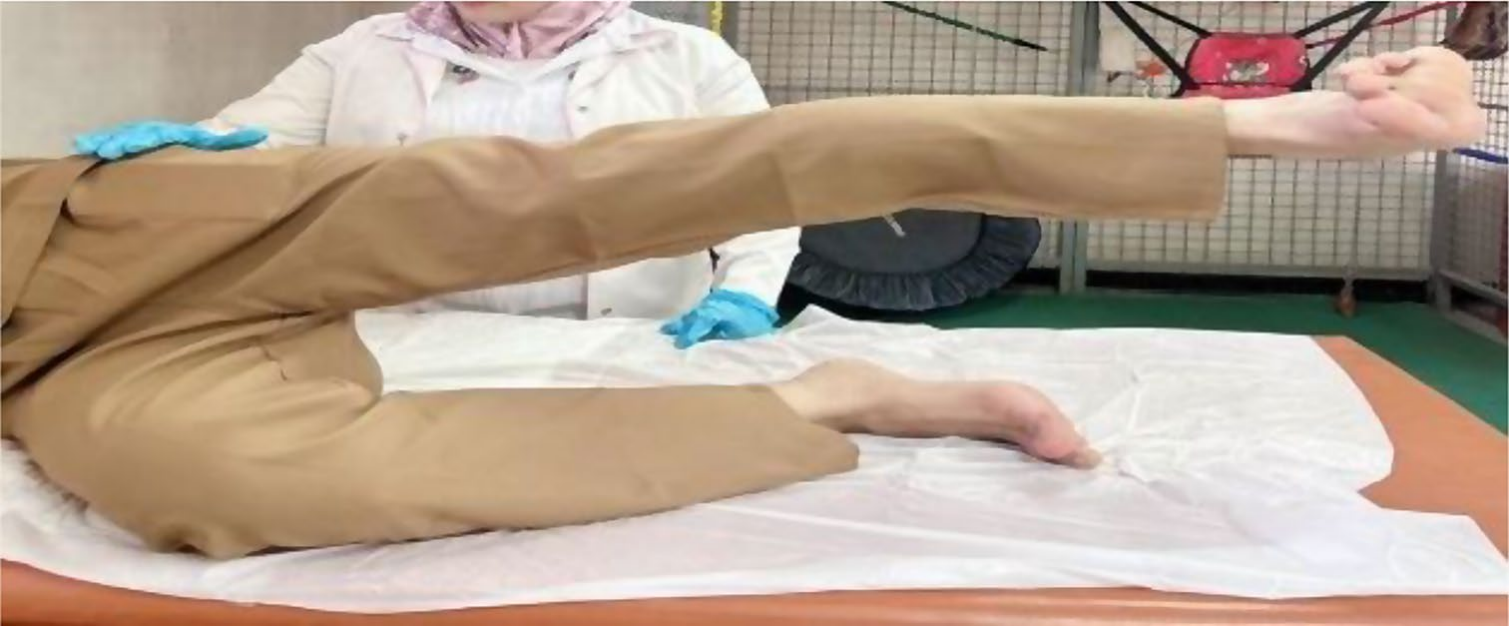
Straight leg raising from side lying (uppermost leg)

**Fig. 6:**
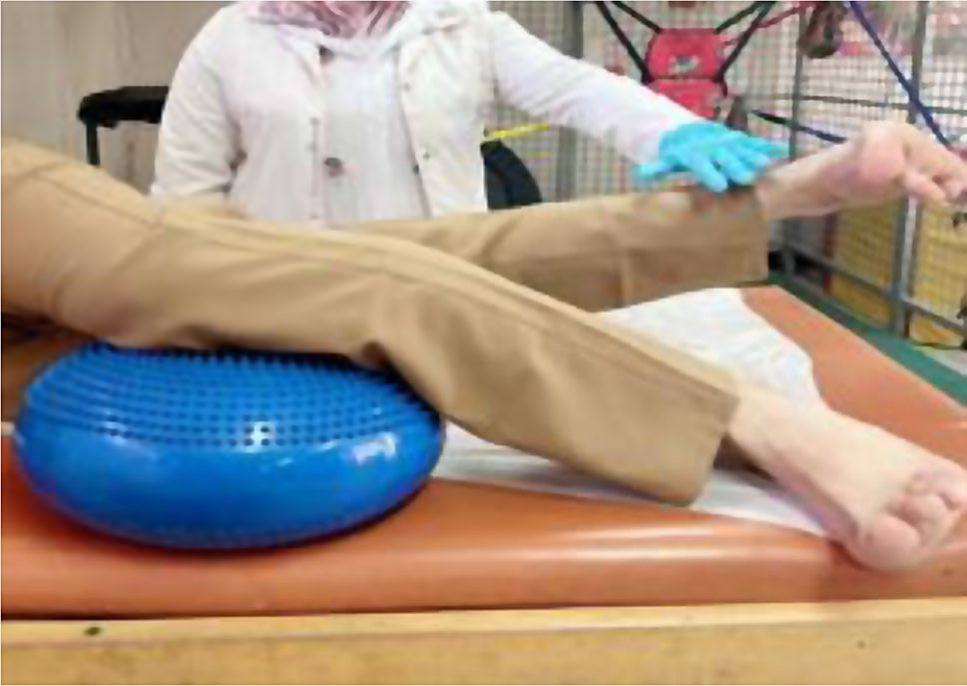
Straight leg raising from side lying (lowermost leg)

Patients in the MFPH + EX group (Group A) were given a combination of MFPH (as in Group B) and a selected exercise program. The exercises for the affected knee joint included supine hamstring stretches, calf stretches (Fig. 1), a terminal knee extension (Fig. 2), and straight leg-raising (SLR) exercises (Figs. 3, 4, 5 and 6). Stretching exercises were performed for 15–20 s each, with 3–5 repeats and rest intervals in between. Strengthening exercises were performed in 2–3 sets of 10 repetitions, with a 5-second hold and a 10-second rest break between repetitions (Appendix).

In all groups (A, B, and C), treatments were given three times per week for four consecutive weeks.

### Statistical analysis

The sample size was determined using the F-test (MANOVA), a repeated measure between factors with 80% power and 5% type I error. The effect size (0.3) was calculated using the primary outcome (WOMAC) from a prior study [12], which found that dexamethasone phonophoresis was clinically and statistically significant in improving KOA pain and function. The minimum sample size was 69; however, it was increased by 15% to accommodate dropouts. The appropriate sample size was 78 participants. G* Power version 3.1.9.2 (Franz Faul, Uni Kiel, Germany) was utilized for the calculations.

Data were evaluated for normality, variance homogeneity, and extreme scores before analysis as a prerequisite for parametric analysis of the variance. There were no outliers in the box plots or whiskers, and the Shapiro-Wilk and Variance’s homogeneity tests showed that the data was normally distributed with variance homogeneity except for VAS.

Descriptive statistics and one-way analysis of variance (ANOVA) were used to examine the demographic characteristics of the three groups, including mean age (years), weight (kg), height (cm), and BMI (kg/m^2^). Simultaneously, a Chi-squared test was used to assess sex distribution among groups. A mixed 3 × 3 design MANOVA evaluated the impact of treatment (between groups), time (pretreatment, 2 weeks post-treatment, and 4 weeks post-treatment), and interaction on mean values of knee ROM and functional capability (function and physical performance). The Kruskal-Wallis test (between groups) and Friedman test for time (pretreatment, two weeks post-treatment, and four weeks post-treatment) were utilized to assess pain intensity. Post-hoc analyses were performed using the Bonferroni correction to adjust for multiple comparisons. The significance level was set to *p* < 0.05. All statistical tests were performed using Version 20 of the Statistical Package for Social Studies (SPSS) for Windows (IBM SPSS, Chicago, IL, USA).

## Results

**Fig. 7:**
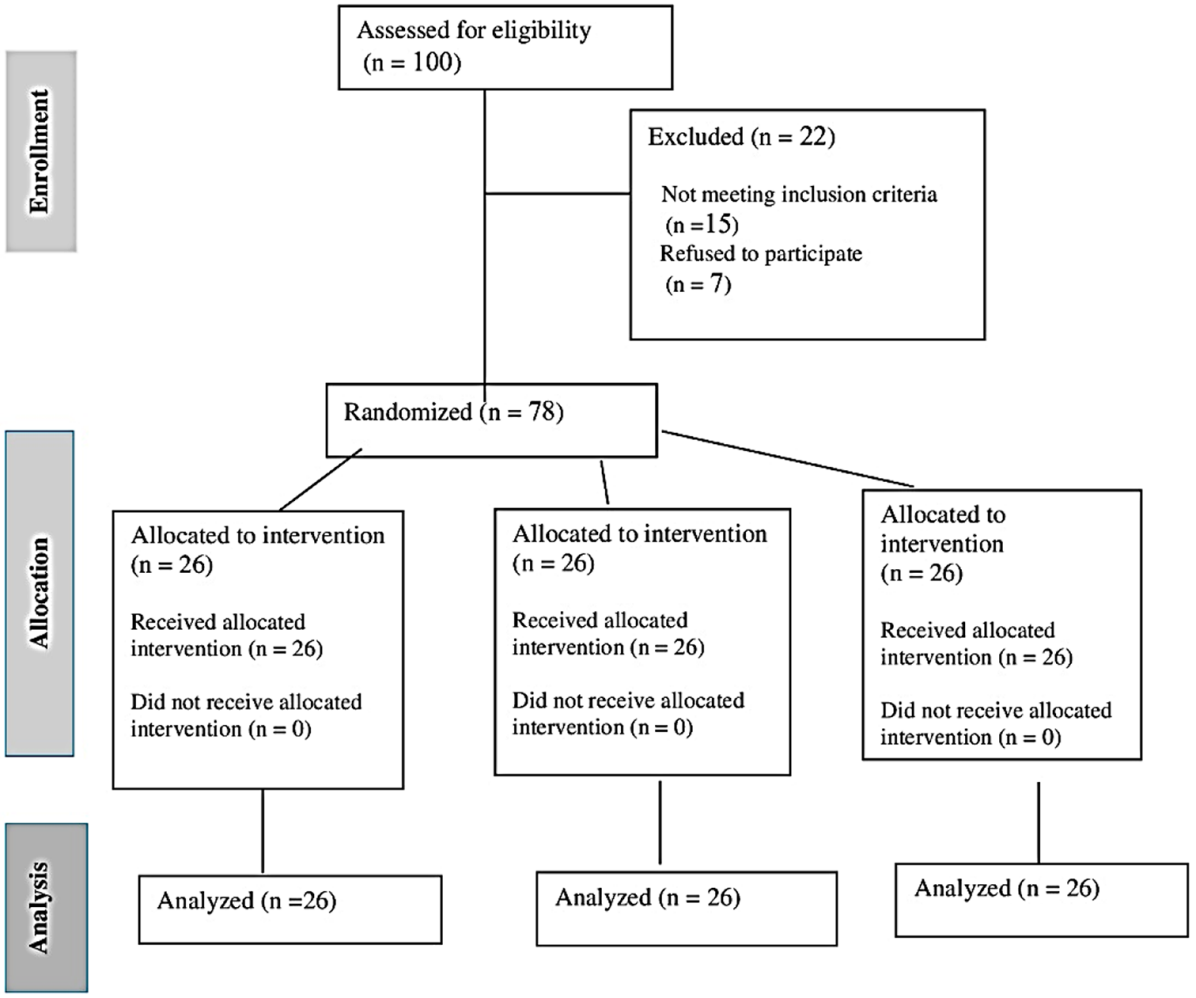
Flow chart for patients’ eligibility

One hundred patients were screened for eligibility. Fifteen patients did not match the inclusion criteria, and seven declined to participate (Fig. 7). Demographic data analysis revealed no statistically significant differences between the three groups in mean values of age, gender, weight, height, BMI, and lower limb dominance (*p* > 0.05). The data normality analysis using the Shapiro-Wilk test revealed that all variables had a normal distribution except VAS. Table 1 summarizes the demographic features of the patients.

**Table 1:** General characteristics of subjects of three groups

Subject characteristics	Group A (MFPH + EX)	Group B (MFPH)	Group C (US)	f-value	p-value
**Age (years)**	53 ± 6.9	54 ± 6.4	54.2 ± 7.6	0.213	0.809
**Weight (kg)**	78.5 ± 9.8	76.4 ± 5.8	73.3 ± 7.3	2.84	0.064
**Height (cm)**	167.6 ± 10.8	166.2 ± 7.8	164.6 ± 8.6	0.702	0.449
**BMI (kg/m** ^**2**^ **)**	27.9 ± 1.8	27.8 ± 1.2	27 ± 1.5	2.24	0.113
**Sex** **Male** **Females**	12 (46%) 14 (54%)	10 (40%) 16(60%)	11 (43%) 15 (57%)	**χ**^**2**^ **=** 0.315	0.854
**Dominance** **Right** **Left**	14 (54%) 12 (46%)	14 (54%) 12(46%)	11 (43%) 15 (57%)	**χ**^**2**^ **=** 0.923	0.630

Data expressed as mean ± SD: standard deviation, **N**: number, **%**: (percentage), **χ**^**2**^: chi-square, **P value**: level of significance, **MFPH + EX**: metformin phonophoresis, and selected exercise, **MFPH**: metformin phonophoresis, **US**: ultrasound with non-drug standard coupling gel

Regarding the overall effect of treatment on measured variables, MANOVA revealed a significant main effect of time and (treatment*time) (*p* = 0.001) and a significant effect of treatment (*p* = 0.005) (Table 2).

**Table 2:** MANOVA table for the effect of treatment on the measured variables

Interaction effect (treatment * time)	p-value	ƞ^2^
* F = 6.15*	*p* = 0.001*	*0.420*
**Effect of time**		
* F = 157.6*	*p* = 0.001*	*0.949*
**Effect of treatment**		
* F = 2.87*	*p* = 0.005*	*0.137*

**F value**: level of Variance, **P value**: level of significance, ƞ^**2**^: partial eta square, *: Significant

### Knee pain

At the pretreatment measurement, no statistically significant difference was seen between the three groups (*P* = 0.994). On the other hand, statistically significant differences in mean VAS values were reported in both the 2-week and 4-week post-treatment conditions (*P* = 0.023, *P* = 0.001, respectively) (Table 3). At 2 weeks post-treatment, groups A and B showed statistically significant improvement compared to group C (*P* = 0.013; MD: -1; CI: -1.8 - -0.1, *P* = 0.022; MD: -0.8; CI: -1.6–0.1, respectively). At 4-week post-treatment, groups A and B showed statistically significant improvement compared to group C (*P* = 0.001; MD: -1.6; CI: -2.3 - -0.9, *P* = 0.001; MD: -1.2; CI: -1.9 - -0.4, respectively) (Table 4).

**Table 3:** Comparison between pretreatment, 2-week post-treatment, and 4-week post-treatment mean values of VAS, WOMAC, knee flexion, and extension ROM between and within groups

Measured variables	Group A (MFPH + EX)	Group B (MFPH)	Group C , (US):	f-value	P value	ƞ^2^
**VAS** (cm)				**χ** ^**2**^		
Pre-study	7.3 ± 1.7	7.5 ± 1.1	7.5 ± 1.2	0.012	0.994	0.005
2- week post-treatment	4.9 ± 1.5	5.1 ± 1	5.9 ± 1.3	7.58	0.023*	0.1
4- week post-treatment	2.8 ± 1.2	3.2 ± 0.8	4.4 ± 1	23	0.001*	0.29
(P-value)	0.001*	0.001*	0.001*			
**WOMAC** Pre-study	48.3 ± 11.2	51.5 ± 8.5	50.3 ± 14.4	0.473	0.625	0.012
2- week post-treatment	30.5 ± 12.7	37.7 ± 9.1	42.3 ± 14	6.36	0.003*	0.145
4-week post-treatment	17.3 ± 12	24.6 ± 8	34.4 ± 13	14.6	0.001*	0.281
(P-value)	0.001*	0.001*	0.001*			
**Knee flexion (degrees)** Pre-study	60 ± 7.3	68.5 ± 7.4	64.7 ± 7	2.72	0.073	0.068
2-week post-treatment	73 ± 7.5	71 ± 8.4	66.7 ± 7.2	4.54	0.014*	0.108
4-week post-treatment	77.2 ± 7.7	74.7 ± 9.2	68.6 ± 7.6	7.55	0.001*	0.168
(P-value)	0.001*	0.015*	0.197			
**Knee extension (degrees)** Pre-study	72 ± 7.3	72.7 ± 6.7	68.6 ± 6.6	2.68	0.075	0.067
2- week post-treatment	75.5 ± 6.7	75.8 ± 7.3	70.4 ± 6.6	5.02	0.009*	0.118
4- week post-treatment	80 ± 6.9	79.8 ± 8	72.6 ± 7.1	8.56	0.001*	0.186
(P-value)	0.001*	0.002*	0.125			

Data is represented as mean ± SD, **WOMAC**: Western Ontario and McMaster Osteoarthritis Index, **VAS**: visual analogue scale, *: significant, ƞ^**2**^: partial eta square, **MFPH + EX**: metformin phonophoresis and selected exercise, **MFPH**: metformin phonophoresis, **US**: US with non-drug standard coupling gel, **χ**^**2**^: chi-square

**Table 4:** Post hoc test between groups of knee pain and function

Post hoc between groups		WOMAC		VAS	
		2-week post-treatment	4-week post-treatment	2-week post-treatment	4- week post-treatment
Group A vs. B	MD (95% CI) P-value	-7.1 (-15, 1) 0.105	-6.7 (14.5, 1) 0.116	-0.2 (-1, 0.7) 0.855	-0.4 (-1.1, 0.3) 0.156
Group A vs. C	MD (95% CI) P-value	-11.7 (-19.8, -3.6) 0.002*	-17.1 (-24.9, -9.3) 0.001*	-1 (-1.8, -0.1) 0.013*	-1.6 (-2.3, -0.9) 0.001*
Group B vs. C	MD (95% CI) P-value	-4.6 (-12.7, 3.5) 0.503	-10.4 (-18, -2.6) 0.005*	-0.8 (-1.6, 0.1) 0.022*	-1.2 (-1.9, -0.4) 0.001*

MD: mean difference, CI: confidence interval, vs.: versus, *: significant, **WOMAC**: Western Ontario and McMaster Osteoarthritis Index *: significant, **VAS**: Visual analogue scale

When comparing time effects on the three groups, it was noticed that all groups A, B, and C had statistically significant changes in pain reduction (*P* = 0.001) (Table 5).

In all groups, A, B, and C, the order of significant changes was pre vs. 4-week post-treatment, followed by 2-week vs. 4-week post-treatment, and pre vs. 2-week post-treatment (*P* = 0.001). However, it should be highlighted that the maximum improvement was documented after 4 weeks post-treatment in all groups A (*P* = 0.001; MD:4.5; CI: 4–4.9), B (*P* = 0.001; MD:4.3; CI:3.8–4.7), and C (*P* = 0.001; MD:3.1; CI: 2.6–3.5) (Table 5).

### Physical function (WOMAC index)

At the pretreatment measurement, no statistically significant difference was reported between the three groups (*P* = 0.0625). On the other hand, statistically significant differences in mean values of WOMAC were reported in both 2-week and 4-week post-treatment conditions (*P* = 0.003, *P* = 0.001), respectively (Table 3). At 2- week post-treatment, the results revealed significant improvement in group A compared to group C (*P* = 0.002; MD: -11.7; CI: 19.8 - -3.6) and no statistically significant improvement in group A compared to group B (*P* = 0.105; MD: -7.1 CI: -15–1) and in group B compared to group C (*P* = 0.503; MD: − 4.6; CI: -12.7- 3.5) (Table 4). At 4-week post-treatment, the results revealed significant improvement in groups A and B compared to group C (*P* = 0.001; MD: -17.1; CI: -24.9- -9.3, *P* = 0.005: MD: -10.4: CI: -18- -2.6, respectively) (Table 4).

When comparing time effects on the three groups, it was noticed that all groups A, B, and C showed statistically significant changes in WOMAC mean values (*P* = 0.001) (Table 5). For group A, the order of substantial changes was (pre vs. 4-week post-treatment, pre vs. 2-week post-treatment, and 2-week versus 4-week post-treatment) (*P* = 0.001), in addition to similar findings were noticed in group B and group C (*P* = 0.001, *P* = 0.001). However, it should be noted that maximum improvement was reported after 4-week post-treatment in group A and similar findings in group B, followed by group C (*P* = *0.001; MD: 31; CI: 27- 34.8*,*P* = *;0.001; MD: 27.5; CI: 23.7–*31, *P* = 0.001; MD:15.8; CI: 12- 19.6, respectively) (Table 5).

### Knee flexion ROM

At the pretreatment measurement, no statistically significant difference was reported between the three groups (*P* = 0.073). On the other hand, statistically significant differences in mean values of knee flexion ROM were reported in both 2-week and 4-week post-treatment conditions (*P* = 0.014, *P* = 0.001), respectively (Table 3). At 2-week post-treatment, the results revealed early significant improvement in group A compared to group C (*P* = 0.013; MD: 6.3; CI: 1-11.5) and no statistically significant improvement in group A compared to group B and group B compared to group C (*P* = 1; MD: 1.9; CI: -3.3- 7.11, *P* = 0.129; MD: 4.4; CI; -0.8-9.7, respectively). At 4-week post-treatment, the results revealed significant improvements in groups A and B compared to group C (*P* = 0.001; MD: 8.6; CI: 3- 14.1, *P* = 0.027; MD: 6; CI: 0.5–11.6, respectively) (Table 6). When comparing time effects on the three groups, it was noticed that no statistically significant change in knee flexion ROM was reported in group C (*P* = 0.197). Otherwise, statistically significant differences were seen in groups A and B (*P* = 0.001, *P* = 0.015, respectively) (Table 3).

**Table 5:** Post hoc test between groups of knee flexion and extension ROM

Post hoc between groups		Knee flexion		Knee extension	
		2- week post-treatment	4-week post-treatment	2- week post-treatment	4- week post-treatment
Group A vs. B	MD (95% CI) P-value	1.9 (-3.3, 7.1) 1	2.5 (-3, 8) 0.822	-0.3 (-5, 4.2) 1	0.3 (-4.7, 5.3) 1
Group A vs. C	MD (95% CI) P-value	6.3 (1, 11.5) 0.013*	8.6 (3, 14.1) 0.001*	5 (0.4, 9.7) 0.030*	7.5 (2.5, 12.5) 0.001*
Group B vs. C	MD (95% CI) P-value	4.4 (-0.8, 9.7) 0.129	6 (0.5, 11.6) 0.027*	5.4 (0.7, 10.1) 0.017*	7.2 (2.2, 12.2) 0.001*

MD: mean difference, CI: confidence interval, vs.: versus, *: significant, ROM: range of motion

**Table 6:** Post hoc test between measurement times of pain and function

Post hoc between measures		WOMAC			VAS		
		Group A	Group B	Group C	Group A	Group B	Group C
Pretreatment vs. 2 weeks post-treatment	MD (95% CI) P-value	17.8 (14.5, 21) 0.001*	13.8 (10.5, 17) 0.001*	8 (4.7, 11.3) 0.001*	2.4 (2, 2.7) 0.001*	2.4 (2, 2.7) 0.001*	1.6 (1.3, 1.9) 0.001*
Pretreatment vs. 4 weeks post-treatment	MD (95% CI) P-value	31 (27, 34.8) 0.001*	27.5 (23.7, 31) 0.001*	15.8 (12, 19.6) 0.001*	4.5 (4, 4.9) 0.001*	4.3 (3.8, 4.7) 0.001*	3.1 (2.6, 3.5) 0.001*
2 weeks post- treatment vs. 4 weeks post-treatment	MD (95% CI) P-value	13.3 (11.6, 15) 0.001*	13.6 (12, 15.3) 0.001*	7.8 (6.2, 9.5) 0.001*	2.1 (1.8, 2.4) 0.001*	1.9 (1.6, 2.2) 0.001*	1.5 (1.2, 1.8) 0.001*

MD: mean difference, CI: confidence interval, vs.: versus, *: significant, **WOMAC**: Western Ontario and McMaster Osteoarthritis Index, vs.: versus, *: Significant

The order of significant changes was (pre versus 4-week post-treatment, 2-week versus 4-week post-treatment, and pre versus 2-week post-treatment (*P* = 0.001) for group A, in addition to similar findings that were noticed in group B (*P* = 0.001). However, it should be highlighted that maximum improvement was reported after 4 -weeks post-treatment in group A and then in group B with a mean difference (*P* = 0.001 MD: -8.1; CI: -13.3-2.9, *P* = 0.001; MD: -6.2; CI: -11.4, -1, respectively) (Table 7).

**Table 7:** Post hoc test between measurement times of knee ROM (flexion, extension)

Post hoc between measures		Knee flexion		Knee extension	
		Group A	Group B	Group A	Group B
Pretreatment vs. 2 weeks post-treatment	MD (95% CI) P-value	-3.9 (-9, 1.3) 0.001*	-2.6 (-7.8, 2.6) 0.001*	-3.4 (-8.2, 1.3) 0.001*	-3.1 (-7.9, 1.6) 0.001*
Pretreatment vs. 4 weeks post-treatment	MD (95% CI) P-value	-8.1 (-13.3, -2.9) 0.001*	-6.2 (-11.4, -1) 0.001*	-8 (-12.8, -3.3) 0.001*	-7 (-11.8, -2.3) 0.001*
2 weeks post- treatment vs. 4 weeks post-treatment	MD (95% CI) P-value	-4.2 (-9.4, 0.9) 0.001*	-3.6 (-8.8, 1.6) 0.001*	-4.6 (-9.3, 0.1) 0.001*	-3.9 (-8.6, 0.8) 0.001*

MD: mean difference, CI: confidence interval, vs.: versus, *: significant, ROM: range of motion

### Knee extension ROM

At the pretreatment measurement, no statistically significant difference was reported between the three groups (*P* = 0.075). In contrast, statistically significant differences in mean values of knee extension ROM were reported at 2-week and 4-week post-treatment conditions (*P* = 0.009, *P* = 0.001), respectively (Table 3). At 2- week post-treatment, the results revealed significant improvement in groups A and B compared to group C (*P* = 0.030; MD:5; CI:0.4–9.7, *P* = 0.17; MD:5.4; CI: 0.7–10.1, respectively) (Table 6). At 4-week post-treatment, the results revealed significant improvement in groups A and B compared to group C (*P* = 0.001; MD: 7.5; C: I2.5-12.5, *P* = 0.001; MD:7.2; CI:2.2–12.2, respectively) (Table 6).

When comparing time effects on the three groups, group C had no statistically significant change in knee extension range of motion (*P* = 0.125); otherwise, statistically significant differences were detected in groups A and B (*P* = 0.001, *P* = 0.002), respectively (Table 6).

The order of significant changes was (pre vs. 4-week post-treatment, 2 weeks vs. 4-week post-treatment, and pre vs. 2-week post-treatment (*P* = 0.001) for group A; comparable results were also observed for group B (*P* = 0.001). However, it should be noted that maximum improvement was reported after 4 weeks of treatment in group A and then in group B with mean differences (*P* = 0.001; MD: -8; CI: -12.8 -3.3, *P* = 0.001; MD; -7; CI; -11.8- -2.3, respectively) (Table 7).

## Discussion

This study aimed to assess the potential benefits of metformin delivery by phonophoresis in conjunction with exercise therapy compared to either MFPH alone or conventional US treatment (with non-drug acoustic gel) on knee pain, physical function, and flexion and extension ROM in patients with mild to moderate unilateral KOA. The findings revealed that the MFPH + EX and MFPH groups significantly improved in the clinical measures (knee pain, function, and ROM) after the 4-week intervention compared to the conventional US group. However, MFPH improved clinical outcomes, particularly when combined with exercises. These findings could be explained by the therapeutic advantages of the hypothesis that metformin has a regulatory effect on chondrocytes (chondroprotective), reduces knee articular cartilage loss, and has anti-inflammatory and antioxidant effects [16, 33–37]. Consequently, metformin may slow down the progression of osteoarthritis.

Concerning knee pain, the study found that following a 2-week intervention, the MFPH + EX and MFPH groups experienced an early reduction in knee pain compared to the US group. This finding could be attributed to metformin’s analgesic, anti-inflammatory, and antioxidative properties [36–38]. The delivery of metformin directly to the affected joint by phonophoresis may result in a more targeted and potentially effective method of reducing osteoarthritis pain. The US group experienced pain reduction after 4 weeks of treatment, attributed to the US’s thermal and mechanical properties facilitating tissue healing. However, to be effective, it has to be used for more than 4 weeks [10].

Notably, in all groups, the most substantial reduction in pain was observed after a 4-week intervention; thus, it is recommended that MFPH treatment alone or in combination with exercise be continued for a longer time (more than four weeks) to maximize the benefits of the intervention.

Regarding physical function, all groups showed improvement after 2-week and 4-week interventions. Nevertheless, the 4-week intervention showed the most remarkable improvement, indicating that MFPH should continue longer. It was observed that following a 2-week intervention in group A (MFPH + EX), there was an early improvement and reduction of WOMAC scores. This finding clarifies the impact of exercises as a cornerstone in managing KOA. The evidence suggests that exercises promote joint function, reduce pain, and improve patients’ overall quality of life with KOA [5, 6, 39]. As a result, when combined with MFPH, exercises may have better and earlier effects than when used alone.

This study’s findings are consistent with earlier research emphasizing the benefits of metformin’s analgesic and anti-inflammatory properties, which, in turn, enhance physical function in KOA patients, with efficacy increasing with time of treatment. It has been demonstrated that oral metformin (2 g/day) for 24 months resulted in changes in the WOMAC score and VAS value [21]. A previous trial reported that taking metformin (1000 mg/day) for 12 weeks in combination with meloxicam enhanced the knee injury and osteoarthritis outcome score (KOOS) components [38].

Compared to oral methods, local metformin administration via phonophoresis into the knee joint could boost local medication absorption and minimize adverse reactions [40, 41]. The superior improvement of pain and function in the MFPH + EX group can be attributed to the synergistic effects of the combined intervention. Phonophoresis, which improves the transdermal delivery of metformin gel, likely provided a more immediate reduction in inflammation and pain. The combined exercise program contributed to pain reduction and enhanced patient functionality by improving joint function and muscular strength, and it probably promoted improved metformin absorption through enhanced blood flow and tissue mobility [5, 6, 39, 41]. As a result, this highlights the potential benefits of combined MFPHs and exercises as a multimodal treatment strategy for KOA.

In terms of knee flexion ROM, group A (MFPH + EX) demonstrated an early and substantial improvement after a 2-week intervention, and both group A (MFPH + EX) and group B (MFPH) experienced a significant improvement after a 4-week intervention. However, groups A (MFPH + EX) and B (MFPH) showed equivalent improvement in extension ROM during the 2-week and 4-week interventions, respectively. This improvement emphasizes the combined effects of MFPH and exercises in clinical settings, which may improve knee flexion ROM early. This study found that US treatment had no significant impact on knee ROM, indicating that improved ROM outcomes can be attained by combining US application with other therapeutic modalities.

The findings align with earlier studies that demonstrated the beneficial effects of phonophoresis using various medicinal products to treat KOA. Numerous studies have shown that phonophoresis is beneficial in reducing pain and issues related to physical function in patients with KOA [42, 43]. These effects include reduced blood levels of inflammatory indicators, increased antioxidant levels [43], improved pain intensity, and enhanced functional mobility [12]. However, it should be noted that these studies used different medicinal products. In terms of pain relief and improved function, Phonophoresis with certain drugs, such as piroxicam [15] and diclofenac sodium [44] showed to be more effective than US treatment alone However, there were no significant differences between US and phonophoresis with other medications [14, 44–47]. Combined exercises and phonophoresis resulted in better outcomes, as observed with the Zingiber Cassumunar Roxb treatment [48]. The dissimilarities observed between the findings and those of other clinical trials could be attributed to using different phonophoresis agents, subject selection based on different Kellgren-Lawrence indexes, and applying various US parameters (power, frequency, or duration) and trial duration. To the greatest extent of the author’s knowledge, no clinical trials of MFPH in patients with musculoskeletal disorders have been conducted despite several studies comparing the efficacy of US and phonophoresis with anti-inflammatory medications in KOA.

Based on the results, it can be claimed that subcutaneous circulation using phonophoresis picks up considerable amounts of metformin, and MFPH + EX effectively improves KOA symptoms (pain level, limited ROM, and impaired physical functions). The claim of penetration to depths of several centimeters has been validated in clinical practice through systematic reviews and meta-analyses. According to the analysis, phonophoresis may account for up to 30% of physiotherapy visits. Approximately 75% of the analyzed research found that the US is an effective booster for topically applied drugs as a treatment for KOA [40–42]. Given the chronic and often debilitating nature of KOA, the study’s findings emphasize the potential for metformin phonophoresis plus exercise to be a more successful treatment approach than either metformin phonophoresis or US therapy alone. Clinicians may consider employing a multimodal strategy with KOA patients to improve therapeutic outcomes.

Despite the positive results, this study has some possible limitations, such as the outcome assessor’s blinding and the short follow-up time. Future research with larger sample sizes, more diverse populations, and more extended follow-up periods is needed to confirm the long-term effects of MFPH and its combination with exercise in KOA management. Future studies may consider the efficacy of MFPH at various doses and with different US modes in OA patients, particularly diabetic patients, as well as compare the effects of different drugs utilized in phonophoresis with varying exercise regimes.

## Conclusion

Metformin phonophoresis combined with exercise treatment may benefit patients with KOA by improving knee pain, mobility, and functional disability management. These findings support the integration of MFPH into KOA rehabilitation programs, emphasizing the benefits of combining therapeutic modalities and exercise to accomplish optimal patient outcomes. However, our findings may contribute to ongoing research on treating patients with KOA.

### Clinical implications

- Metformin phonophoresis combined with conventional knee exercises can be an effective treatment option for patients with OA, providing earlier pain relief and improved functional capabilities.
- These findings have implications for clinicians and healthcare providers involved in managing KOA, as MFPH can offer a non-invasive and effective treatment option for pain relief, improved function, and improved disease prognosis in this patient population.
- A multimodal approach to treating KOA is more significant than a single intervention.

## Electronic supplementary material

Below is the link to the electronic supplementary material.

Supplementary Material 1

## Data Availability

No datasets were generated or analysed during the current study.

## Abbreviations

KOA: knee osteoarthritis
OA: Osteoarthritis
ROM: Range of motion
VAS: Visual analogue scale
MFPH: Metformin phonophoresis
EX: Exercises
WOMAC: Western Ontario and McMaster Universities Osteoarthritis Index
SLR: Straight leg raising
MANOVA: Multivariate Analysis of Variance
