# Supplementary material for: Effects of metformin phonophoresis and exercise therapy on pain, range of motion, and physical function in chronic knee osteoarthritis: randomized clinical trial
Source: J Orthop Surg Res. 2024 Oct 26;19:689. doi: 10.1186/s13018-024-05120-0 (PMC11515155; doi:10.1186/s13018-024-05120-0)
Supplement: Supplementary file 1 — Supplementary Material 1 [file 13018_2024_5120_MOESM1_ESM.docx]

**Appendix 1**

**Exercise regimen employed in group A of patients with knee osteoarthritis.**

| **Exercise** | **Dose and frequency** |
| --- | --- |
| **Stretching exercise** | |
| Supine hamstring and calf stretch | 15-20 seconds stretch for 3-5 repetitions with rest intervals in between. |
| **Strengthening exercise** | |
| Terminal knee extension | 2-3 sets of 10 repetitions with 5-s hold and 10-s rest interval in between repetitions. |
| Straight leg raising from Supine lying position. | 2-3 sets of 10 repetitions with 5-s hold and 10-s rest interval in between repetitions. |
| Straight leg raising from side-lying position.  (upper leg raising) | 2-3 sets of 10 repetitions with 5-s hold and 10-s rest interval in between repetitions. |
| Straight leg raising from side-lying position.  (lowermost leg raising) | 2-3 sets of 10 repetitions with 5-s hold and 10-s rest interval in between repetitions. |
| Straight leg raising from a prone-lying position.  (upper leg raising) | 2-3 sets of 10 repetitions with 5-s hold and 10-s rest interval in between repetitions. |
